# Mandates are not enough: placement provider and systems factors that drive ambulatory healthcare use for children in foster care

**DOI:** 10.3389/fped.2026.1830287

**Published:** 2026-08-26

**Authors:** Sarah J. Beal, Adam C. Carle, Constance A. Mara, Courtney B. Dunn, Katie Fox, Mary V. Greiner

**Affiliations:** 1 Department of Pediatrics, College of Medicine, University of Cincinnati, Cincinnati, OH, United States; 2 Behavioral Medicine and Clinical Psychology, Cincinnati Children's Hospital, Cincinnati, OH, United States; 3 General and Community Pediatrics, Cincinnati Children's Hospital, Cincinnati, OH, United States; 4 James M. Anderson Center for Health Systems Excellence, Cincinnati Children's Hospital, Cincinnati, OH, United States; 5 Department of Psychology, College of Arts and Sciences, University of Cincinnati, Cincinnati, OH, United States

**Keywords:** child welfare, foster care, healthcare use, linked data, mandated care

## Abstract

**Introduction:**

In the United States, children are mandated to receive healthcare when they enter foster care and regularly thereafter; states may require more frequent visits. Despite regulations and public insurance to support access to healthcare for young people in foster care, health outcomes for this population remain poor. This study seeks to understand the extent to which caregiver/placement characteristics, child characteristics, and an information-sharing intervention are associated with receipt of mandated healthcare visits for youth in foster care, which may help the field to better understand why health disparities persist for youth in foster care.

**Methods:**

This retrospective observational study uses administrative data from the child welfare system linked to the electronic health record at a freestanding children's hospital that is contracted to provide mandated healthcare visits for children entering foster care or experiencing placement changes. All children (0–21 years) in foster care for at least one day in a licensed, kinship, group home, or independent living placement in a single Ohio county between 2012 and 2025 (*N* = 24,904 placements for *N* = 10,729 youth) were included. Cross-classified multilevel logistic models predicted mandated visits within placements and accounted for the cross-classification of children within families of origin, placement providers, and the clustering of repeated observations within children.

**Results:**

The odds of completing a mandated visit was 73%. Placement providers accounted for the largest amount of variance in receipt of mandated care (44% in the adjusted model). Placement in settings other than licensed foster homes (i.e., kinship, group home, independent living) significantly reduced the likelihood of visits. While prior placement providers' experience was generally beneficial, it did not increase visit completion among those in kinship care. Across child characteristics (e.g., age in years, sex, race, ethnicity, number of unique medical diagnoses, number of unique mental health diagnoses), mental health diagnoses were significantly and meaningfully associated with completed visits. Finally, information sharing between healthcare and child welfare systems was associated with a greater likelihood of completing mandated visits (OR = 1.50).

**Discussion:**

These results indicate that simply mandating visits is insufficient to ensure their completion. Caregiver factors, information sharing between healthcare and child welfare systems, and children's needs likely all represent mechanisms by which the healthcare and child welfare systems can increase mandated healthcare use.

## Introduction

In the United States, children are required to receive healthcare when they enter foster care and regularly thereafter – commonly referred to as mandated healthcare visits (1). Local jurisdictions may require mandated healthcare visits at additional frequencies, which align with recommendations from the American Academy of Pediatrics (AAP) and Child Welfare League of America (CWLA) to offer healthcare early (i.e., within 72 h of entry into foster care) and frequently (i.e., twice as often as children would typically be seen for well child exams) to ensure indicators of maltreatment, acute and chronic disease, and developmental delay are detected and intervened upon early (2). Of note, AAP and CWLA recommended visits are not mandated (required) unless state or local jurisdictions also require them. Without requirements, those increased visits are recommendations that healthcare systems and caregivers may follow at their discretion.

Healthcare systems are motivated to offer increased visits to ensure that 1) all children in foster care have access to healthcare that will improve health, and 2) children's services, who have legal guardianship, and caregivers, who are responsible for the day-to-day health of children in foster care, are adequately supported to address the specific needs of each child in foster care. For children's services agencies, ordinances mandating healthcare visits are designed to ensure strong connections to healthcare services for children in foster care, addressing important aspects of child wellbeing. Mandated and more frequent primary and preventive visits are especially important in supporting caseworkers and caregivers because, in pediatric healthcare, the parent or caregiver serves as the gatekeeper, historian, and advocate for their child (3, 4). The separation of a child from their family of origin results in a disconnect that can contribute to gaps in information and care delivery that have the potential to compromise children's health, even beyond the health needs a child might have as a result of abuse or neglect (5). Healthcare access, when combined with Medicaid eligibility for children in foster care (6) and care coordination offered through Medicaid Managed Care Organizations (7) should lead to better health outcomes for children in foster care. Mandated healthcare visits play a critical role in achieving these regulatory goals. Unfortunately, there has been little research on compliance with mandated visits and what factors are associated with the completion of mandated visits for children in foster care, making it difficult to determine how effective mandates are for improving the health of children in foster care.

### The need to connect children to healthcare professionals

Children who enter foster care have often faced household barriers and other challenges that may have prevented them from accessing healthcare services, or might have contributed to healthcare that was episodic and inconsistent (8, 9). These barriers can include issues with insurance, transportation availability, or limitations in healthcare service availability (10–12). As a result, children enter foster care with variable prior histories with healthcare systems. As children enter foster care, the responsibility for ensuring healthcare use transitions from the family of origin to the state or county that holds temporary legal custody of the child, and this responsibility is further regulated through routine federal review of public children's services agencies (13). Mandated visits, especially when delivered by healthcare service systems designed for children in foster care, can support children's services agencies in complying with federal and local Children's Services regulations about healthcare access and use for children in foster care (14).

Children in foster care are more likely to receive a full range of acute diagnoses (15–17), chronic and complex chronic medical diagnoses (18, 19), and mental and behavioral health diagnoses (20–22) than children not in foster care. Children's service staff (23, 24) and placement providers [i.e., licensed foster and kinship caregivers, group home staff, independent living program staff (25–27)] report that they receive insufficient information about children's health needs and histories at the time children in foster care enter placement. Similarly, healthcare professionals often lack complete health histories for children, who may have visited a variety of healthcare systems prior to entry into foster care and over the course of multiple placements while children are in foster care (26, 28). Mandated healthcare visits therefore provide an opportunity for healthcare providers to obtain a complete health history and evaluate the child's current health status, which can be communicated to placement providers, children's services staff, Guardians ad Litem and Court-Appointed Special Advocates, and the family of origin. These visits may result in action plans to manage diagnoses, connect children to ongoing primary care, or connect them to specialty care services for specific diagnoses and conditions. Following recommendations from the American Academy of Pediatrics (29), mandated healthcare visits for children in foster care often include physical exams with laboratory screening (30), immunization record reconciliation with child immunization catch-up when needed, vision and hearing screening, dental screening, screenings for developmental delays and behavioral concerns, and trauma symptoms screening and diagnostic assessments to guide ongoing mental health treatment services (29). Healthcare providers also gather health information from electronic health records, sometimes across healthcare systems (31, 32), which can fill gaps in health histories for children when they are removed from their families of origin or when they change from one placement to another. This can only occur in the context of a healthcare encounter; healthcare systems generally do not know when their patients come into foster care or experience placement changes (28). Mandated visits, therefore, give healthcare systems the opportunity to reconnect with patients.

Automated information sharing is a mechanism by which both healthcare and child welfare systems might improve mandated visit completion and, more generally, children's health outcomes. Automated information sharing uses secure data exchange to link child welfare and healthcare records and display limited, critical information for caseworkers and healthcare providers to access to guide care and service delivery decisions (28, 33). This can include making healthcare systems aware of which children require mandated healthcare services, along with updated contact information for new caregivers to support outreach to get mandated appointments scheduled (26). It can also provide documentation to children's services to ensure mandated visits occur and to help children's services with independent and direct access to health data, rather than relying on caregiver reports of second-hand information (23, 34). Automated data sharing between healthcare and child welfare systems has been shown to increase completion of mandated visits (33) and improve communication between healthcare and child welfare systems (35). However, studies examining automated information sharing have not accounted for other contextual factors (e.g., caregiver behaviors, children's health needs).

### Placements and caregivers

The day-to-day responsibilities of managing children's health needs, including scheduling and bringing children to healthcare visits, rest with the temporary placement provider. These caregivers are also expected to coordinate and communicate with children's services staff who ensure a complete health history and a plan for meeting children's health needs is documented and communicated to the family of origin and the court (25). Barriers to transportation, availability of services, and other challenges faced by caregivers of children in foster care may contribute to gaps in compliance with mandated visits. Additionally, placement providers who are unaware of children's health needs, upcoming appointments, or mandated visit requirements may fail to seek healthcare for children. As caseworkers rely on placement providers for visit completion (23), understanding the unique contribution of placement providers (caregivers and staff) is an important and understudied aspect of children's health needs.

It is also possible that children's services staff and placement providers alike are more likely to adhere to requirements for healthcare visits when children have more medical or mental and behavioral health diagnoses, because those mandated visits provide an opportunity to re-establish care or connect with other services and supports. Thus, children's services, placement providers, family of origin, and child factors might contribute to variability in the healthcare children receive, even when visits are mandated. Understanding these dynamics may help to inform interventions that address known disparities in healthcare access and health outcomes for children in foster care (36, 37).

### Rationale for the current study

In summary, multiple factors contribute to access to and utilization of healthcare for children in foster care, leading to different drivers of healthcare use when children are in foster care compared to other populations of children. As a result, even when children in foster care are insured by Medicaid and healthcare services are required and audited by children's services, some children continue to experience gaps in care and poor health outcomes. The mechanisms contributing to gaps in healthcare are likely driven by placement-level characteristics (e.g., type of placement, familiarity of the caregiver with healthcare visit requirements, placement resources), the unique contexts of the child welfare case (e.g., age of placement, maltreatment history), the unique characteristics of the child (e.g., diagnoses, age, demographics), and the capacity of healthcare and child protection systems to support and ensure children remain connected to care (e.g., information sharing). Whether these factors are important when healthcare visits are mandated (i.e., required by federal or local law) has not been studied. Understanding how these factors interact and uniquely contribute to mandated healthcare use for children in foster care is critical for advancing policy and practice to improve their health and well-being.

### The current study

This study seeks to understand the extent to which placement provider (i.e., caregiver and staff) characteristics, children's services and related experiences, child characteristics, and information sharing between healthcare and children's services are associated with receipt of one or more mandated healthcare visits for youth in foster care during a placement episode. It is expected that placement provider characteristics (e.g., role as licensed, kinship, group home staff; experience with prior placements; level of resources received to support the placement) and youth characteristics (e.g., number of medical and mental health diagnoses, child age, maltreatment history) will be associated with the likelihood that a child receives one or more mandated healthcare visits. Specifically, children placed with licensed foster caregivers and group home staff are expected to have the highest likelihood of receiving visits due to placement provider experience and training. Children placed with kinship caregivers with prior experience caregiving to youth in foster care are also expected to have a higher likelihood of receiving mandated visits. Children with a history of using more healthcare services in general and who are moving to placement providers that receive higher levels of support for caregiving are also expected to have a higher likelihood of receiving mandated visits. We also expect that youth with more diagnoses will be more likely to receive mandated care. Finally, we expect that automated information sharing will increase the likelihood a child will receive mandated care.

## Materials and methods

### Setting

This study was conducted in partnership with Hamilton County Job and Family Services in Ohio and Cincinnati Children's Hospital Medical Center. The Institutional Review Board at Cincinnati Children's Hospital and Prosecutor's office and children's services division at Hamilton County Job and Family Services approved the study. At the time this study was conducted, the state of Ohio required all children entering foster care or experiencing a change of placement to receive a medical evaluation within 5 business days of placement. Hamilton County Job and Family Services contracts with Cincinnati Children's Hospital to complete those exams; as a result, it is expected that all children experiencing entry into foster care or a placement change will receive evaluations through Cincinnati Children's Comprehensive Health Evaluations for Cincinnati's Kids (CHECK) Center (14). The CHECK Center includes integrated services to screen and provide brief intervention and referral to intensive services in the areas of mental health, substance use, oral health, and development. Cincinnati Children's is also the largest primary care network in Hamilton County for children enrolled in Medicaid, the only pediatric provider for most specialty care services, and the only pediatric emergency and inpatient care system in the region. As a result, the bulk of medical care services for children enrolled in Medicaid are provided by Cincinnati Children's.

In 2019, Cincinnati Children's and Hamilton County Job and Family Services partnered to establish IDENTITY (28, 33), a near real-time data exchange platform that allows child welfare professionals to access limited medical information for children in their custody. IDENTITY also allows healthcare providers to know when their patient is in Hamilton County custody (26). This enables the CHECK Center to proactively identify children newly entering foster care or experiencing a change of placement.

### Participants

This retrospective observational study leverages administrative data from the child welfare system linked to one or more electronic health records at Cincinnati Children's Hospital. All children (0–21 years old) in Hamilton County Children's Services custody for at least one day between 2012 and 2025 (*N* = 11,627 youth) were potentially eligible. Most children (98%; *N* = 11,355) had a corresponding electronic health record at Cincinnati Children's and could therefore be included in the study, representing *N* = 29,480 placements. For this analysis, we further restricted to placements in licensed foster homes, kinship homes, group homes, or independent living settings where mandated visits to the CHECK Center would be required, resulting in *N* = 24,904 placements for *N* = 10,729 youth.

### Procedures

Hamilton County Job and Family Services provided identified data to Cincinnati Children's Information Services in March 2025 that reflected all potential participants, consistent with existing policies and regulations for data sharing with IDENTITY. Records were linked by Cincinnati Children's Information Services using matching algorithms and processes that result in deterministic linking of records between Children's Services and the electronic health record (38). Information Services then extracted relevant medical information for all study participants, removed all patient identifiers, and supplied the study team with a limited dataset for analysis while protecting participants' identities.

### Measures

#### Child welfare records

Child welfare records were used to identify placement occurrences, which would indicate the need for a mandated visit. For this reason, the data was structured by placement episode. The child's age at the start of a given placement was calculated using the placement start date and date of birth, expressed in full years (e.g., 0–20). Race was measured as White, Black, American Indian/Alaska Native, Asian, Native Hawaiian/Other Pacific Islander, more than one race, or other race. For the current analyses, we included only White (0) and Black (1) children, as individual race categories other than White and Black accounted for less than 0.5% of the sample and were too sparse to model. Ethnicity was measured as non-Hispanic (0) or Hispanic (1). We used the number of investigated maltreatment allegations to create a time-varying variable that ranged from 0 to 10 or more. Child maltreatment exposure was reported as the total number of substantiated or indicated allegations (captured from 1 to 10 or more for analysis), and also reported by type of maltreatment, coded as 0 (no exposure) or 1 (any exposure) for substantiated or indicated allegations of emotional maltreatment, neglect, medical neglect, physical abuse, and sexual abuse. Placement type was measured as foster home, kinship care, group home, or independent living. For these analyses, we created dummy variables for each of these values with foster home as the reference category. For each placement, the resource allocation (i.e., level of care) provided for the child was reported and used to indicate placement changes to a higher level of care (1; e.g., from a traditional to a treatment foster home), same level of care (0; e.g., from one traditional foster home to another traditional foster home), or move to a lower level of care (−1; e.g., from a group home to a traditional foster home). Finally, prior experience of caregivers was defined as the number of prior placements (0, 1, 2, 3, 4, or 5 or more), excluding placements made concurrently with the target child (i.e., for sibling sets placed with the same provider on the same day).

#### Electronic health records

Our outcome, receipt of mandated care, was coded as 0 for placements with no visits or 1 for placements with any mandated medical visits, as confirmed in the electronic health record. Electronic medical records were also used to indicate child sex coded as 0 (male) or 1 (female), chronic medical conditions coded as the unique number of diagnoses across ICD classification codes, with a count from 0 (no chronic medical conditions) to 3 or more chronic medical conditions, and mental health diagnoses coded using the Children's Hospital Association's Child and Adolescent Mental Health Disorders Classification System (39), with a count from 0 (no mental health diagnoses) to 3 or more mental health diagnoses. Diagnoses occurring at or before a mandated visit for a child were included. Deployment of universal and automated information sharing launched on January 1, 2018, and was reflected in the data as 0 (before data sharing) or 1 (after data sharing).

### Analysis plan

#### Rationale

We used logistic cross-classified multilevel models to estimate the odds of receiving mandated care and the extent to which covariates predicted it (40). We used these models because of the cross-classified nature of the data and the binary outcome. Three classifications were present in the data, and we specified them in our models: child, family of origin (determined via case ID), and placement provider. These are labeled as classifications rather than levels (as in a traditional multilevel model) because they are not strictly hierarchically nested. Repeated observations were possible for each classification. In addition, child and family of origin were cross-classified within placement providers. Specifically, children can have multiple placement and custody episodes (clustering within child), multiple children may be from the same family of origin (clustering within family of origin), or cared for by a single placement provider at a given point in time (clustering within placement provider). In addition, not all children from the same family of origin share the same placement provider (cross-classification within placement providers), and children could have the same placement provider across different points in time (cross-classification across placement providers). Failing to account for a cross-classified structure can result in biased estimates and standard errors. Cross-classified multilevel models appropriately model the dependence arising from a cross-classified data structure and can partition the variance in the outcome attributable to each level (41–44).

#### Model-building procedures

In general, to fit the models, we first ran a given model using an iterative generalized least square (IGLS) algorithm (45, 46), ignoring the cross-classified structure of the data. We then used the estimates from the IGLS model as start values for a given cross-classified multilevel model (47). We estimated all of cross-classified multilevel models using Bayesian Markov Chain Monte Carlo (MCMC) methods and the Metropolis–Hastings algorithm (48). MCMC requires complete cases for analysis. For all models, we followed model diagnostics guidelines described by Singer and Willet (49) to probe for problematic model assumption violations, outliers, or influential points. We conducted all analyses using MLwiN version 2.30 using runmlwin within Stata version 18 (50).

To understand the predictors of mandated care and the proportion of variance accounted for at each level before and after adding predictors, we fit a series of models that followed Singer and Willet (49). The first was a null model. This model included only a random intercept at the child, family of origin, and placement provider levels. We then fit a model with the predictors described above and a random intercept at the child, family of origin, and placement provider levels. In this model (which we label the full model), we specified *a priori* two-way interactions between placement type and (a) child age at placement, (b) total number of prior placements for the provider, and (c) child race, as well as an interaction between child age and race. We include these interactions to evaluate whether the association between placement context and visit completion varied by child characteristics or provider experience. We removed interactions with coefficients near zero for parsimony. Given the large sample size at the observation level (24,904), we focused on effect sizes rather than significance to determine whether to exclude interactions in the final model. We then reran the model (which we label the final model) without interactions that had small effect sizes. We then compared the set of coefficients from the final model to the set of analogous coefficients in the full model to ensure that exclusion of an interaction did not impact the coefficients for the variables that remained. Finally, comparing the random effects from the null and final models allowed us to describe the extent to which the predictors explained the variance that existed in the null model.

Given that these were logistic models, we used the simulation method described by Goldstein to estimate residual-level variance and compute the variance partition coefficient (VPC) for each level (42). In addition, as suggested by Merlo, we used the median odds ratio (MOR), interval odds ratio (IOR), and sorting out index to describe the extent to which variance at each level impacted the odds of mandated care (43, 44). Merlo defines the MOR as the median of the odds ratios between the observation with the highest odds of mandated care and the observation with the lowest odds of mandated care when two observations are randomly selected within a classification (e.g., placement provider). Thus, it describes the extent to which the odds of mandated care would increase if an observation moved to a classification unit (e.g., different placement provider) with a higher odds of mandated care (in the median). The IOR and sorting out index allow one to consider the impact of variation at the child, family of origin, and placement provider levels on predictors. Consider the IOR. Suppose one took all possible pairs of observations with equal covariate values and then computed the odds ratio between the observation in a classification unit with a higher odds of mandated care and the observation in a classification unit with lower odds of mandated care. Merlo et al. define the IOR as the interval that contains 80% of the odds ratio values, centered on the median. The sorting out index gives the percentage of pairs in the distribution of odds ratios in which the odds ratio is greater than 1.

## Results

### Descriptive statistics

Descriptive statistics for the *N* = 10,729 youth included in these analyses are provided in Table 1, using first placement and custody episode for all data that varies over time. Youth were, on average, 7 years old at their first placement, with an average of 2.6 placements over the study period, which ranged from 2012 to 2025. Sex was evenly distributed (49% female), and the racial distribution was fairly balanced (52% Black); the majority of the youth represented were non-Hispanic (97%). Youth had an average of 1 chronic condition and 1 mental health diagnosis. At the time of their first placement, most youth were placed in licensed foster (49%) or kinship homes (45%), followed by group homes (4%) and independent living (2%).

**Table 1:** Descriptive statistics for the study sample at the first placement and custody episode during the study period (2012–2025).

	M (SD) or *N* (%)
Age at first placement	7.11 (5.76)
Sex	
Male	5,473 (51%)
Female	5,256 (49%)
Race	
Black	5,571 (52%)
White	3,902 (36%)
Ethnicity	
Non-Hispanic	10,354 (97%)
Hispanic	368 (3%)
Number of Chronic Conditions	0.86 (1.25)
Number of Mental Health Diagnoses	1.28 (1.86)
Number of Placements per Child	2.63 (2.31)
Number of Allegations	3.49 (3.53)
Type of Allegation	
Any Substantiated Physical Abuse	5,447 (51%)
Any Substantiated Sexual Abuse	428 (4%)
Placement type	
Level of care	0.77 (0.62)
Licensed foster home	5,254 (49%)
Kinship placement	4,768 (45%)
Group home	477 (4%)
Independent living	230 (2%)

### Cross-classified multilevel models

#### Variance and cross-classification

As noted, we first fit a null cross-classified model to estimate the odds of receiving mandated care, with a random intercept at the child, family, and placement provider (caregiver) levels. The null model indicated that the unconditional probability of receiving mandated care was 0.73 ($b_{\mathrm{Null}\;\mathrm{Model}Child,Family,Provider}$ = 1.01 CI = 0.90–1.13; see Table 2).

**Table 2:** Model fit statistics and variance components for models without any predictors (Null Model) and with final predictors (Full Model).

Variance Component	Variance Partition Component Median Odds Ratio (SD)		Interval Odds Ratio (IOR)			
			−80%	+80%	−80%	+80%
	Null Model	Full Model	Null Model		Full Model	
Child	0.358 (0.065)	0.013 (0.023)	0.234	0.509	0.067	0.154
Family of Origin	4.740 (0.313)	2.877 (0.198)	4.173	5.334	2.516	3.290
Label Placement Provider (caregiver)	11.867 (0.703)	8.973 (0.522)	10.587	13.438	8.024	10.056

The variance components of this null model indicated that 46% of the variance in the odds of receiving mandated care was explained by the placement provider (caregiver), 18% by the family of origin, and 1% by the child. Thirty-four percent of the variance occurred within children. The $\mathrm{MO}R_{\mathrm{Provider}}$ for the null model equaled 26.74, and the $\mathrm{MO}R_{\mathrm{Family}\;\mathrm{of}\;\mathrm{Origin}}$ and $\mathrm{MO}R_{\mathrm{Child}}$ equaled 7.98 and 1.77, respectively. Thus, the odds of receiving mandated care increased nearly 27-fold (in the median) if a given child were theoretically able to move from a caregiver with a lower odds of having a child placed with them receive mandated care to a caregiver with a higher odds of having a child placed with them receive mandated care. Similarly, the odds of receiving mandated care increased nearly 8-fold (in the median) if a given child were theoretically able to move from a family of origin whose children had lower odds of mandated care to one whose children had higher odds of mandated care. Finally, the odds of receiving mandated care increased nearly 2-fold (in the median) at the child level.

Next, we fit a cross-classified model (the full model) with each of the variables described in the measures section. With respect to the random effects, the covariates included in the model (covariate effects described in detail below) explained 30% of the variance (across all levels) in the null model (see Table 2). The variance components of the final model indicated that, after accounting for covariates, 44% of the variance in the odds of receiving mandated care was attributable to the placement provider (caregiver) level, 14% to the family of origin level, and <1% to the child level. The $\mathrm{MO}R_{\mathrm{Provider}}$ for the final model equaled 17.41 and the $\mathrm{MO}R_{\mathrm{Family}\;\mathrm{of}\;\mathrm{Origin}}$ and $\mathrm{MO}R_{\mathrm{Child}}$ equaled 5.04 and 1.36, respectively.

#### Caregiver and placement-associated predictors

Table 3 presents the results from the final cross-classified model. As Table 3 shows, all fixed effects in this model were statistically significant (*p* < 0.05). The conditional probability of receiving mandated care was 0.86 ($b_{\mathrm{Final}\;\mathrm{Model}Child,Family,Provider}$=1.71 CI = 1.53–2.02).

**Table 3:** Results of logistic cross-classified multilevel models predicting the completion of mandated healthcare visits, accounting for variation at the level of the specific placement episode, child, family of origin, and placement provider levels.

	M (SD)	OR	Credible Interval	
			−95%	+95%
Intercept	1.78 (0.13)**^a^		1.53	2.02
Child demographics				
Age at the start of Placement	−0.18 (0.01)**	0.83	−0.20	−0.17
Sex (Female = 1)	0.11 (0.06)*	1.12	−0.01	0.23
Race (Black = 1)	**−0.39** (0.09)**	**0**.**68**	**−0**.**55**	**−0**.**21**
Ethnicity (Hispanic = 1)	**1.08** (0.04)**	**2**.**93**	**0**.**62**	**1**.**55**
Chronic Conditions (0, 1, 2, 3 or more)	0.33 (0.04)**	1.39	0.26	0.40
Mental Health Diagnoses (0, 1, 2, 3 or more)	**0.51** (0.03)**	**1**.**66**	**0**.**45**	**0**.**57**
Child maltreatment exposures				
Any Substantiated Physical Abuse	0.13 (0.07)*	1.13	0.00	0.26
Any Substantiated Sexual Abuse	−0.25 (0.14)*	0.78	−0.52	0.02
Total Allegations (0–10 or more)	−0.03 (0.01)*	0.97	−0.06	0.00
Placement provider characteristics				
Move to a Lower Level of Care	0.19 (0.08)*	1.21	0.04	0.36
Move to a Higher Level of Care	0.33 (0.07)**	1.39	0.19	0.49
Prior experience as a placement provider, ranging from 0 to 5 or more prior placements	0.27 (0.02)**	1.30	0.23	0.31
Group home placement type	**−2.83** (0.40)**	**0**.**06**	**−3**.**53**	**−2**.**04**
Independent living placement type	**−0.90** (0.20)**	**0**.**41**	**−1**.**30**	**−0**.**51**
Kinship placement type	**−0.52** (0.10)**	**0**.**59**	**−0**.**72**	**−0**.**32**
Kinship X prior provider experience	**−0.40** (0.05)**	**0**.**67**	**−0**.**50**	**−0**.**31**
Automated Information Sharing	**0.41** (0.08)**	**1**.**50**	**0**.**26**	**0**.**56**

Bolded values indicate findings with meaningful effect sizes.

a The log odds of the intercept (1.78) correspond to a conditional probability of 0.86.

*p < 0.05.

**p < 0.01.

At the provider level, the type of placement had a relatively large effect on the likelihood of receiving mandated care (Table 3). Relative to licensed foster care placements, kinship placements (OR = 0.59), group home placements (OR = 0.06), and independent living placements (OR = 0.41) all decreased the odds of receiving mandated care. In general, a one-unit increase in the number of placements a provider had previously increased the odds that a child would receive mandated care (OR = 1.30). However, for kinship placements, a one-unit increase in the number of placements a kinship provider previously had decreased the odds that a child would receive mandated care (OR = 0.67). Thus, for kinship providers only, the overall impact of prior placement experience is near zero. Finally, whether the current placement provider was receiving a higher (OR = 1.39) or lower (OR = 1.21) level of support relative to the child's previous placement both increased the likelihood a child would receive mandated care.

#### Child-associated predictors

Whether a child was Hispanic or not (OR = 2.93), the number of chronic conditions (OR = 1.39), the number of mental health diagnoses (OR = 1.66), and whether a child was Black or White (OR = 0.68) were the child-level predictors with the largest effect sizes. While the OR for age (OR = 0.83) was not large, given the range of ages among children (0–17), the effect of age could still be substantial.

We initially included two-way interactions between placement type and age, placement type and the total number of placements, placement type and race, and age and race. However, with the exception of an interaction between kinship placement and placement history (a proxy for placement provider experience; b = −0.4, *p* < 0.05), the remaining interaction terms were near zero (e.g., 0.01), and most were not statistically significant. Thus, we kept the interaction with kinship and placement total and dropped the remaining interaction terms in the final model.

#### Information sharing effects

When a child entered a placement with automated information sharing in place, the adjusted odds of completing a mandated visit increased by 50% (OR = 1.50, see Table 3). Given our study's interest in the impact of automated information sharing between healthcare and child welfare systems on the odds of a child receiving mandated care, we present the IOR and sorting out index for information sharing only to preserve space. The $\mathrm{MO}R_{\mathrm{Lower}Child}$ for automated information sharing equaled 0.84, and the $\mathrm{MO}R_{\mathrm{Upper}Child}$ equaled 2.68. Thus, given variation at the child level centered on the median, the 80% interval of the distribution of odds ratios for automated information sharing includes some children who were less likely to receive mandated care when automated information sharing was in place. However, although the interval includes 1.00, the sorting out index shows that for 81% of children who entered care when automated information sharing was in place, the odds of receiving mandated care were positive.

## Discussion

Healthcare use, even when free via Medicaid enrollment and mandated by local regulations, is complicated for children in foster care. In this study, the probability of any mandated visit during a placement was 0.73, suggesting that up to 1 in 4 children in foster care are unlikely to receive mandated visits. The predictors of mandated visit completion are complex and are attributed to child characteristics and placement provider characteristics, described in more detail below. Finally, information sharing between healthcare and child welfare systems was associated with a 50% increase in the relative odds of completing a visit. Notably, establishing automated data sharing between healthcare and child welfare systems benefited approximately 81% of placements in the study after automated data sharing was in place. This is likely because healthcare and child welfare systems are able to effectively identify children in need of mandated visits and contact them more efficiently (26, 35). While more work remains to close the gap in access to medical care, information sharing with outreach to placement providers appears to be a critical and effective component toward that goal and represents something healthcare and child welfare systems can do to improve compliance with federal and local mandates.

### Child characteristics

The relative proportion of variance attributable to the child (e.g., diagnoses, maltreatment history, demographics) was low (<1%), which is clinically relevant because it indicates that efforts to increase compliance with mandated healthcare visits should focus on factors other than the child's characteristics. Further, much of the variance at the child level was accounted for with the covariates included in these analyses, including demographic, maltreatment, and child health constructs. Across those factors, only three contributed meaningfully [OR ≥ 1.5 or <0.75 due to incidence rate of mandated visits (51)]: mental health, race, and ethnicity. Youth with a mental health diagnosis were 66% more likely to complete a mandated visit compared to youth without a mental health diagnosis. The foster care clinic where mandated visits occur offers embedded mental health services (14). Caregivers and child services professionals may be more motivated to ensure visits are completed for children with mental health diagnoses as a way of ensuring they receive behavioral health services. Of note, one might expect a similar phenomenon for youth with physical health concerns; however, that was not the case. Medical diagnoses contributed a modest 39% increase in the likelihood of completing a mandated visit compared with children without a medical diagnosis. This may reflect a system-level emphasis on behavioral health needs in this context compared to physical health concerns or may suggest that accessing mental health services is harder than accessing physical health services, motivating completion of mandated visits where those services can be initiated.

With respect to race and ethnicity, Hispanic youth were nearly 3 times more likely to complete mandated visits than non-Hispanic children, while Black youth were 32% less likely to complete mandated visits than White children. Black youth are more likely to be placed in kinship (52, 53) and congregate care settings (54), which may contribute to lower mandated visit completion rates. Notably, the coefficients for race-by-placement-type interactions were near zero, and their inclusion or exclusion did not affect the other coefficients in the model. This suggests more complexity in race-based factors driving mandated healthcare use beyond placement type. With respect to ethnicity, Hispanic youth make up a relatively small proportion of the overall population of children in foster care in our region (3%), so effects for Hispanic youth will need to be replicated in other communities.

#### Clinical impact of child-related findings

These results suggest that race-based differences in compliance with mandated visits exist and are not explained by placement setting. Understanding why those differences persist may help explain disparities in health outcomes for Black youth in foster care (55). Mental health may also be a reason that young people complete mandated visits, although this could be related to the inclusion of behavioral health in mandated visits at this clinic site.

### Caregivers and placement providers

Most of the variance in predicting completion of mandated visits was at the placement provider level (44%), where both the type of placement and the number of prior placements a provider had contributed meaningfully to the likelihood of completing visits, and the effects of level of support were not as large. The large amount of variance highlights placement providers as an important area of focus for efforts to increase compliance with healthcare service mandates. Furthermore, most of the variance at the placement provider level remained unexplained. This suggests that factors beyond what is routinely captured in administrative data (e.g., placement type, level of care, experience) are likely the key contributors to further reducing gaps in healthcare use for children in foster care – warranting additional study and intervention.

With respect to placement type, youth in group homes were 94% less likely than youth in licensed foster care placements to complete mandated visits, while youth in independent living were 59% less likely and youth in kinship placements were 41% less likely compared to licensed foster care placements. This suggests that children in non-family settings are most vulnerable to missing the mandated visits that healthcare and child welfare systems rely on to improve child health. Placement provider experience generally increased mandated visit completion; however, for kinship caregivers, it did not. While all placement providers may benefit from education about the importance of mandated visit completion specifically and the health service needs of children in foster care more generally, kinship caregivers might especially benefit from additional education at the time of placement about mandated visits, along with other supports that can reduce barriers to engagement in healthcare. This would help to ensure more equitable outcomes for all children in foster care, regardless of placement type.

#### Clinical impact of placement provider findings

It is notable that this is, to our knowledge, the first study to examine healthcare use in a way that accounts for the cross-classified nature of children in foster care across families of origin, placements, and time. That nearly half of the variance in the receipt of mandated visits was accounted for by the placement provider is remarkable, and may be indicative of the sizeable role of placement providers in altering how healthcare is used by children while they are in foster care, which has largely been ignored in the existing literature examining healthcare service use for children in foster care [e.g., (21, 37, 56)], including by the authors of the current paper [e.g., (22, 57)]. In addition to attending to placement providers generally, these findings suggest that focusing on preventive healthcare use among youth in non-family settings and on targeted outreach to kinship caregivers may be especially important for increasing compliance with mandated visits. There are a variety of different factors that could be attributed to caregivers and may explain variance across placement settings, including training and knowledge about mandates regarding healthcare access for youth in foster care, transportation and communication barriers, familiarity with healthcare systems access for children in foster care, and access to case management and other services that would help maintain engagement with healthcare systems. Whether these mechanisms explain variation in placement provider effects needs to be explored, as those differences associated with better engagement in mandated care may be ideal targets for intervention.

### Need for future research

Two-thirds (0.66) of the variance in mandated visit completion was allocated at the child, family of origin, and placement provider levels, suggesting that up to 1/3 of the reasons mandated care occurred were unrelated to child, caregiver, or family history factors. Knowing what these factors are and how they can be influenced should be a future focus of studies examining how healthcare and child welfare systems use mandated visits to improve child health outcomes. Second, more research specifically focused on placement provider supports and interventions is clearly needed, given the prominent contribution of placement providers to the overall variance in predicting healthcare visits (44%) required by child welfare policy in this study. There was insufficient data to explain why group homes and independent living programs, for example, were less likely to comply with mandated visits, which could be a focus of future research. Third, following a similar analytic approach to examine healthcare use in other forms (e.g., behavioral health care, primary care, emergency and inpatient care) and the impact of placement provider variation is needed to guide researchers, healthcare systems, and policy-makers in considering the influence of placement providers in addressing what has been a high-cost and largely intractable challenge in health and social service systems.

### Limitations

There are several important limitations to the current study. First, the data is restricted to a single healthcare system and county children's services agency, so generalizability to other healthcare systems and child welfare agencies needs to be established. Second, given the large amount of variance at the placement provider level, there are likely placement provider-level factors (e.g., challenges with caregiver employment or transportation demands) and other characteristics (e.g., incorrect contact information recorded) that are not currently captured in the data. This missing data limits our capacity to fully predict the reasons for mandated visit completion when relying solely on administrative records, which are designed for regulatory and billing purposes rather than explanatory modeling, and thus likely omit contextual, relational, and structural factors that shape healthcare engagement in foster care. Additionally, variation attributed to placement providers may partially reflect differences in documentation practices, caseworker enforcement, or healthcare system workflows rather than purely caregiver behavior. Studies of this kind, conducted alongside surveys or similar designs to capture more complete data, might enhance these models. Third, because automated information sharing was implemented at a specific point in time, its estimated effect may be partially confounded with other secular changes in policy, workflow, staffing, or healthcare delivery occurring during the study period. Fourth, due to small cell sizes, racial categories other than Black and White were excluded from multivariable models, limiting our ability to examine differences (positive or negative) across the full range of racial and ethnic identities represented in foster care. We also lacked information on placement providers' locations, limiting our ability to examine whether placement provider effects were specific to certain geographic regions or features (e.g., distance to hospital). Finally, as an observational study, these findings reflect associations and should not be interpreted as causal effects.

In spite of these limitations, this study substantially advances the field's understanding of the dynamics that support collaboration between healthcare and child welfare systems and its impact on children's healthcare use, with a focus on children in foster care. Mandated healthcare visits for children in foster care aim to close healthcare gaps and support the well-being of these children. Our findings demonstrate that simply mandating visits is insufficient to ensure their completion. Placement providers are the primary source of variance in the odds of completed visits. This indicates a critical role for placement provider training and support, especially for kinship, independent living, and group home placements. Finally, automated information sharing between healthcare and child welfare systems provides mechanisms by which the healthcare system can proactively engage with children and families, and was associated with increased visit attendance; these results suggest it is an important part of visit completion.

## Data Availability

The raw data supporting the conclusions of this article will be made available with reasonable request and appropriate data sharing agreements with Cincinnati Children's Hospital and Hamilton County Job and Family Services.
